# A Signaling Network for Patterning of Neuronal Connectivity in the Drosophila Brain

**DOI:** 10.1371/journal.pbio.0040348

**Published:** 2006-10-10

**Authors:** Mohammed Srahna, Maarten Leyssen, Ching Man Choi, Lee G Fradkin, Jasprina N Noordermeer, Bassem A Hassan

**Affiliations:** 1 Laboratory of Neurogenetics, Department of Molecular and Developmental Genetics, Flanders Interuniversity Institute for Biotechnology (VIB), University of Leuven School of Medicine, Leuven, Belgium; 2 Laboratory of Developmental Neurobiology, Department of Molecular Cell Biology, Leiden University Medical Center, Leiden, Netherlands; Howard Hughes Medical Institute, United States of America

## Abstract

The precise number and pattern of axonal connections generated during brain development regulates animal behavior. Therefore, understanding how developmental signals interact to regulate axonal extension and retraction to achieve precise neuronal connectivity is a fundamental goal of neurobiology. We investigated this question in the developing adult brain of Drosophila and find that it is regulated by crosstalk between Wnt, fibroblast growth factor (FGF) receptor, and Jun N-terminal kinase (JNK) signaling, but independent of neuronal activity. The Rac1 GTPase integrates a Wnt-Frizzled-Disheveled axon-stabilizing signal and a Branchless (FGF)-Breathless (FGF receptor) axon-retracting signal to modulate JNK activity. JNK activity is necessary and sufficient for axon extension, whereas the antagonistic Wnt and FGF signals act to balance the extension and retraction required for the generation of the precise wiring pattern.

## Abstract 2

This neurodevelopment study dissects how development of axonal wiring in theDrosophila optic lobe is regulated by crosstalk between Wnt, fibroblast growth factor receptor (FGFR), and Jun N-terminal kinase (JNK) signaling.

## Introduction

Determining how axon growth is regulated is important, both for our understanding of how the brain is wired during development, and to help devise therapeutic strategies to regenerate damage or diseased neural tissues. The ability of axons to navigate their environment and find their appropriate targets has been divided into several consecutive steps [1–4]. First, neurons sprout neurites, one of which subsequently becomes an axon and extends. Axons then navigate through a maze of negative and positive cues to their appropriate targets. Mistargeted axons must be retracted or degraded. Finally, axons recognize their targets and switch from extension to branching to make functional synaptic contacts. The processes of growth cone formation, axon pathfinding, target recognition, and synapse formation have received much attention. Not surprisingly, these complex processes require a large number of genes and pathways [5–7].

That extension of axons, per se, may be regulated independently from pathfinding is suggested by several observations. Most pathfinding mutants cause axons to change their navigational routes rather than arrest prematurely [8,9]. Conversely, mutations in genes like *short stop, chickadee, dfmr1,* and the small GTPases *Rho, Rac,* and *Cdc42* have been shown, in various contexts, to either antagonize or promote neurite extension [10–12]. The Rho GTPases have been demonstrated to act as signal transducers in a number of signaling pathways [13,14] and play well-documented roles in controlling neurite sprouting, extension, retraction, and guidance in vertebrates and invertebrates [15,16]. Despite important advances in our understanding of the genetic control of axon projection, much remains to be learned. In particular, it not yet clear how various simultaneous, and sometimes antagonistic signals are integrated to result in the appropriate extension or retraction of an axon. Furthermore, whether or not neuronal activity is required for neurite extension during development has been debated [17,18]. Finally, it is interesting to investigate how the behavior of individual axons translates into stereotyped wiring patterns of whole populations of neurons.

We studied a group of cells in the fruit fly brain called the dorsal cluster neurons (DCNs). DCNs are ~40 clustered neurons located in the dorso-lateral central brain. They are part of the Drosophila adult visual system and innervate the optic lobes [19]. DCN axons form a highly stereotyped pattern of connections between two areas of the fly optic lobes called the lobula and the medulla. This regular pattern enabled us to perform a systematic genetic dissection of the requirements for, and interactions of, signal transduction pathways during connectivity patterning in the adult fly brain. We carried out a candidate gene search for modulators of the normal pattern of DCN axon projections within the medulla. We found that Wnt and fibroblast growth factor (FGF) signals modulate Jun N-terminal kinase (JNK) phosphorylation levels, and it is therefore likely that JNK activity, via Rac1, regulates the extension and retraction of DCN axons.

Wnt signaling plays important roles in many cellular process including cell fate, adhesion, and polarity in various developmental contexts [20–23]. Recently, Wnt signaling has been found to play a role in axon guidance and fasciculation in studies of cultured neurons, flies, and vertebrates [24–26,60]. Similarly, the FGFR (fibroblast growth factor receptor) pathway is essential for various developmental processes [27] and has been shown to interact with Wnt signaling in limb development, inner ear development, neural induction, and cell migration [28–30]. In addition, neurite outgrowth assays and injury recovery models suggest that growth factors like FGF and nerve growth factor regulate neurite outgrowth, guidance, and regeneration in culture [31–35]. However, whether such effects are specific to neurite outgrowth or secondary to the enhanced survival and growth effects is unclear. The JNK pathway [36–39] plays a role in cell movement and cell death, possibly by regulating Actin dynamics [40].

We dissected the roles of these pathways and their interactions during axonal extension. Our data suggest that DCN axons extend in a JNK-dependent manner. Upon arriving at their target area, these axons are exposed to a FGF Branchless (Bnl) retraction signal requiring the Breathless (Btl) FGFR. Btl signaling results in the activation of Drosophila Rac1, in turn suppressing JNK activity. While most axons begin to retract, 11–12 are stabilized by a Wnt-mediated signal acting via the Frizzled (Fz) receptors and their adaptor protein Disheveled (Dsh). Wnt signaling results in the suppression of Rac1 activity, thus permitting the JNK extension pathway to remain active. Single-cell mutant analyses suggest that these signals act cell-autonomously. In summary, the adult pattern of DCN connectivity is established, at least in part, through the Rac1/JNK-mediated integration of two simultaneous antagonistic signals.

## Results

### Development of the DCN Connectivity Pattern

**Figure 1:**
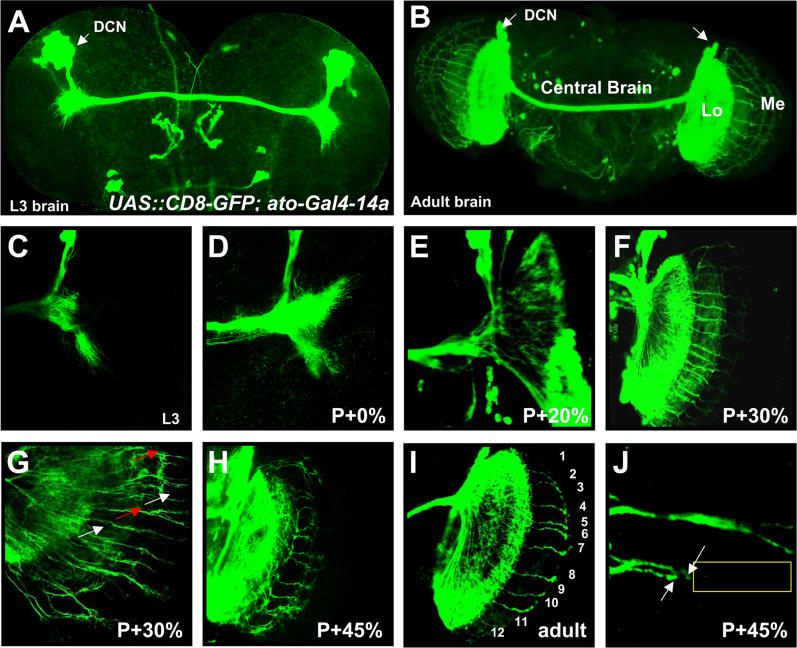
Development of the DCN Axon Connectivity Pattern (A) Dorsal view of a L3 brain from a *w; UAS::CD8GFP/+; ato-Gal4-14a/+* animal. GFP expression is detected in two clusters of ~40 neurons (arrow) with a commissure bridging the two hemispheres. A bundle of axons extends from each commissure into the developing optic lobes. (B) Confocal section of a whole-mount adult brain from a *w; UAS::CD8GFP/+; ato-Gal4-14a/+* fly stained for GFP. Expression is detected in the two clusters (arrows). The commissure is visible, as is the elaborate innervation of the optic lobes by the DCNs. (C) Confocal section of a right hemisphere of a L3 brain, showing that DCNs extend a bundle of axons ventrally through the brain. (D) Confocal section of right hemisphere of a pupal brain (P + 0%), showing the extension of DCN axons toward the developing optic lobes. (E) Confocal section of a right hemisphere of a pupal brain (P + 20%) revealing DCN commissural axons shortly before they extend across the optic chiasm into the medulla. (F) Confocal section of a right hemisphere of a pupal brain (P + 30%). Approximately 30 axons are detected traversing the optic chiasm. (G) A magnification of the ventral half of a similar brain to (F). Note that there are two types of axons: thick, regularly spaced (red arrows) and thin (white arrows). Many of the thinner axons are seen retracting back toward the lobula. (H) Confocal section of a right hemisphere of a pupal brain (P + 45%), the thin axons crossing at P + 30% have retracted leaving 12–14 axons. (I) Confocal section of a right hemisphere of an adult brain; the final structure is now established with 12 axons extending toward the distal medulla. (J) Crossing axons between the lobula and the medulla from a four-cell DCN clone. No evidence for axonal blebbing or fragmentation is seen in the area vacated by the retracting axons (yellow rectangle).

The DCNs are a group of ~40 neurons located dorso-laterally in each brain hemisphere. They are higher-order visual system neurons which innervate the lobula and the medulla neuropils of the Drosophila optic lobes. Their innervation of the optic neuropil originates from a supra-esophageal commissure clearly visible in third instar larval (L3) brains (Figure 1A). The DCNs form extensive branches in the adult lobula. In the medulla, branches originate from 11–12 parallel axons which have crossed the second optic chiasm between the lobula and medulla (Figure 1B). DCN axon extension into the optic lobes takes place during metamorphosis between L3 and the adult stages, making the DCNs an attractive model in which to study the development and control of axon extension directly in the developing brain.

We first characterized the development of this pattern between L3 and adult stages. The DCNs can be visualized and specifically perturbed at these stages through the use of the *atoGal4-14a* line, a UAS-controlled reporter gene and any other transgene of interest. This Gal4 line is expressed in the DCNs beginning in early L3 shortly before they begin to extend their axons toward the optic lobes [19] and continues to express in the DCNs during metamorphosis and into adult life. Importantly, this driver is not expressed in the DCNs during early larval development when their precursors are dividing or during the formation of their commissure. Therefore, the use of the *atoGal4-14a* driver enabled us to evaluate candidate genes acting specifically during DCN innervation of the optic lobes. Transgenes encoding the membrane-associated CD8-green fluorescent protein (GFP) or cytoplasmic β-Galactosidase (β-Gal) were used to visualize DCN axons. In L3 brains (Figure 1A) DCN axons have already formed a commissure that begins sprouting growth cones bilaterally toward the developing optic lobes at puparium formation (PF) (Figure 1C–1H). At approximately 20% PF (Figure 1E), contralateral DCN axons innervate the lobula and form a dense front of fibers at the junction between the lobula and the medulla. At least 30 of these axons project toward the medulla by approximately 30% PF (Figure 1F). Many of these axons have disappeared by 45% PF (Figure 1H). Between 50% and 70% PF, the number of axons crossing the optic chiasm between the lobula and medulla reaches the final number still detectable after eclosion (11–12 with an average of 11.7 axons/hemisphere; Figure 1I).

Axons that do not remain in the medulla might either retract to the lobula or degenerate in place as has been demonstrated to occur during the neuronal remodeling in other systems [41,42]. To test this possibility, we carefully examined small clones of wild-type DCN axons at various stages during pupal brain development. We did not find any evidence for axonal blebbing or fragmentation (Figure 1J). In addition, we generated and evaluated more than 30 wild-type single DCN clones for axon fragment formation but were not able to detect such fragments. These data support the likelihood that the DCN axons that do not innervate the medulla retract back to the lobula.

**Figure 2:**
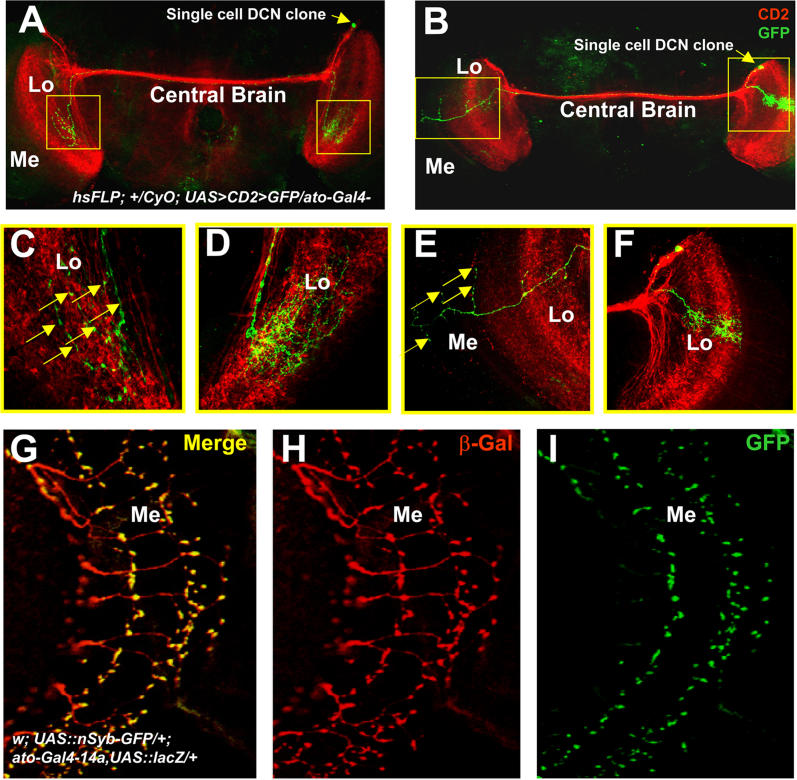
DCN Cells Innervate the Distal Medulla with Contralateral Axons Confocal section of whole-mount adult brains from a *hsFLP; +/CyO; UAS>CD2>GFP/ato-Gal4-14a* (heat shocked at 37 °C for 2 min during L3) stained for GFP (green) and CD2 (red). (A and B) Expression of CD2 is detected in the two clusters, GFP stains a single DCN cell (arrow) which shows contralateral and ipsilateral projections. (C) High magnification of the contralateral projection of the cell shown in (A), corresponding to an axon branching within the lobula. The branches show a typical bouton-like morphology suggesting a pre-synaptic terminal (arrows). (D) High magnification of the ipsilateral projection of the same cell. It branches within the lobula and forms dense, dendrite-like trees. (E) High magnification of the contralateral projection of the cell shown in (B), corresponding to an axon which extends toward the medulla and also has the typical bouton-like morphology in the distal medulla (arrows). (F) High magnification of the ipsilateral projection of the same cell. It branches within the lobula and forms dense, dendrite-like trees. (G–I) Confocal sections of pre-synaptic terminals from (*w; UAS::nSyb-GFP/+; ato-Gal4-14a,UAS::lacZ/+*) adult brains stained for β-Gal (red in H) and GFP (green in I). (G) Colocalization (yellow) of the β-Gal and nSyb-GFP in this terminal indicating that the contralateral projections are axons.

Wild-type adult flies show little variation in the number of DCN axons extending between the lobula and medulla: 71.4% of the flies examined had 12 axons/hemisphere, whereas 28.6% had 11 (*n* = 28). To analyze DCN axons in greater detail, we further examined wild-type DCN clones (Figure 2A–2F). Analysis of single-cell DCN clones generated via Flipase (FLP) mediated recombination using either the FLP-out [43] or MARCM (mutant analysis using a repressible cell marker) techniques [20] shows that contralateral commissural DCN axons have two alternative choices. The majority, corresponding to axons which retracted during pupal development, branches within the lobula (Figure 2A and 2C). The rest of the axons extend toward the medulla and form terminal branches in the distal medulla (Figure 2B and 2E). Quantitative analysis of single-cell clones reveals that approximately 63% (12/19) of the wild-type DCN axons remain in the lobula and 37% (7/19) cross the second optic chiasm to the medulla. The ipsilateral branches of both kinds of clones remain within the lobula and form dense, dendrite-like trees (Figure 2D and 2F). That these contralateral extensions are likely axons is suggested by three observations. First, it has been shown that τ-LacZ, a fusion protein frequently used as an axonal marker, localizes to these neurites [19]. Second, the terminal branches of these neurites have a typical bouton-like morphology, suggesting they bear presynaptic apparatus (Figure 2E). Finally, we find that the presynaptic marker, nSyb-GFP [44], accumulates specifically in these terminals (Figure 2G–2I). Using different markers, a recent report [45] comes to very similar conclusions.

Taken together, these data provide the following picture of the evolution of the DCN connectivity pattern during adult brain development. Initially all DCN axons extend toward the developing medulla. However, during metamorphosis only 11–12 axons are stabilized along specific paths while the intervening axons retract. This raises a number of questions. First, what are the roles of intrinsic versus extrinsic factors in DCN axon outgrowth? Second, is the choice between extension and retraction determined strictly cell-autonomously or do extrinsic factors play roles? If the latter is correct, what signals cause most, if not all of the DCN axons to extend early in development? Third, what signals cause many of these axons to retract later? Fourth, how are the 11–12 axons crossing the optic chiasm at the end of pupal development stabilized? Finally, how are these different signals integrated to produce the adult pattern?

### Identifying Pathways Required for DCN Extension and Retraction

To begin to address these questions we used a candidate gene approach to search for modifiers of the number of DCN axons crossing between the lobula and the medulla (Table 1). We assayed for statistically significant changes in the number of DCN axons spanning the optic chiasm in the absence of obvious effects on the structure of the DCN commissure, DCN axon guidance, cell number, position, and overall morphology. To perform the screen, we initially used the *atoGal4-14a* driver together with UAS::CD8-GFP and UAS-driven transgenic inhibitors of key signal transduction pathway members. We selected transgenes that specifically result in well-characterized loss of function phenotypes for the genes they target.

**Table 1:**
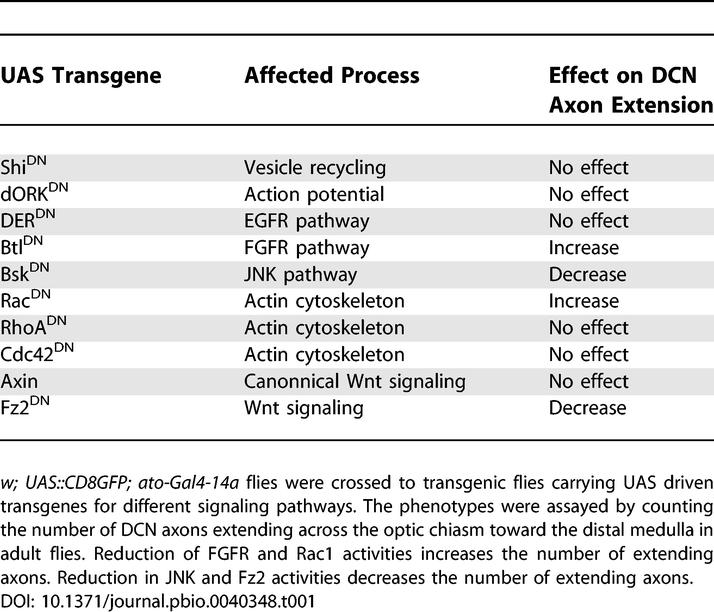
The Effects of Transgenes Expressed in the DCNs on Axonal Extension to the Medulla

We found that DCN axon extension was not affected by blocking neuronal activity via expression of an activated potassium channel [46] or by blocking vesicle recycling through inhibition of Drosophila Dynamin [47] (Table 1). These data suggest that neuronal activity is not required for DCN axon extension, in agreement with largely normal brain development observed in mice lacking Munc18–1, a protein essential for neurotransmitter release [18]. Similarly, blocking the activities of the epidermal growth factor (EGF) receptor [48], RhoA [49], CDC42 [50], and canonical Wnt signaling [51] had no effect on the final number of DCN axons crossing the optic chiasm (Table 1). However, blocking JNK activity [52] or the Fz2 receptor [53] significantly decreased the number of DCN axons crossing between the lobula and the medulla, suggesting that these genes are required for DCN axon extension, stabilization, or both. In contrast, blocking the activities of the FGFR Btl [54] or the Rac1 GTPase [50] significantly increased the number of DCN axons crossing between the lobula and the medulla, suggesting that these genes are required for DCN axon retraction (Table 1).

### JNK Signaling Is Essential for DCN Axon Extension

**Figure 3:**
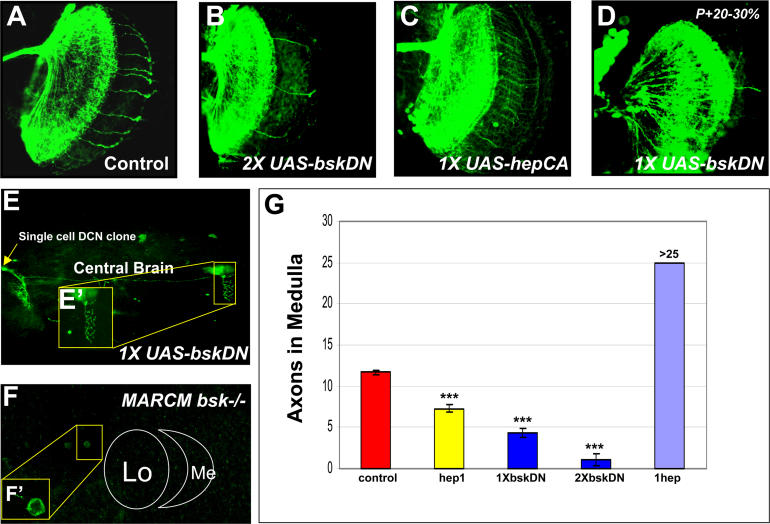
JNK Signaling Is Required for DCN Axon Extension (A) Confocal section through a *w; UAS::CD8GFP/+; ato-Gal4-14a/+*. Normal axon extension from the lobula to the medulla is observed. (B) Confocal section through a brain from *UAS::bskDN/Y; UAS::CD8GFP/+; ato-Gal4-14a/+* showing a reduction in the number of axons crossing the optic chiasm. (C) Confocal section through a *w; UAS::CD8GFP/+; ato-Gal4-14a/UAS::hepA* adult brain, a large increase in the axons crossing the optic chiasm is observed. (D) Confocal section through a *UAS::bskDN/+; UAS::CD8GFP/+; ato-Gal4-14a/+* at P + 20%–30% showing that the reduction of the number of axons crossing observed with *bskDN* occurs early during development. (E) Confocal section of a whole-mount adult brain from a *UAS::bskDN/hsFLP; +/CyO; UAS>CD2>GFP/ato-Gal4-14a* fly stained for GFP. The single DCN cell shows contralateral and ipsilateral projections. (E') High magnification of the branching in the lobula of the contralateral side showing no obvious defect in branching and in the bouton-like morphology. (F) Confocal section of a whole-mount adult brain showing single *bsk* MARCM mutant DCNs obtained from *hs-FLP; FRT40, tubGAL80/bsk^2^, FRT40; ato-Gal4-14a,UAS::lacZ/+* animals with no axonal outgrowth. (F') High magnification of a single *bsk* mutant DCN. (G) Quantification of the axonal extension phenotype of the different genotypes shown in (A–D). A total of at least ten samples were evaluated for each genotype, axons were counted from each, and the average calculated (*** *p* < 0.001)

To further address the role of JNK signaling in DCN axon extension, we counted the number of DCN axons crossing the second optic chiasm in mutants for the fly Jun N-terminal Kinase Kinase (JNKK) *hemipterous (hep)*. In flies homozygous for the hypomorphic *hep^1^* allele, an average of only 7.3 axons innervated the medulla. To further evaluate the requirement of JNK signaling during DCN innervation of the medulla, we expressed one or two copies of a dominant-negative form of Drosophila JNK *basket* (Bsk-DN) in the DCNs, specifically during the phase when they innervate the optic lobes using the *atoGal4-14a* driver. Bsk-DN was previously shown to phenocopy *bsk* loss-of-function mutations during embryogenesis [55]. Expression of one copy of Bsk-DN results in the reduction of the number of DCN axons crossing toward the medulla from an average of 11.7 axons in wild-type flies (Figure 3A) to an average of 4.4 axons (Figure 3G). Expression of two copies of Bsk-DN (Figure 3B) almost completely blocks axon crossing (an average of 1.1) with 30% showing no axon crossing at all. These data suggest that JNK is required for DCN axon extension toward the optic lobes. We then evaluated whether increased levels of JNK signaling are sufficient to force axons to remain extended by expressing a constitutively active Hep transgene [52] in the DCNs. This resulted in all DCN axons crossing to the medulla and none of them retracting, even in 3- to 5-d-old adult brains (Figure 3C). These data support the hypothesis that JNK signaling is necessary and sufficient for all DCN axons to extend toward the medulla.

The reduction of DCN axons crossing the chiasm when JNK signaling is blocked may result either from a failure in their initial extension or from excessive retraction. To distinguish between these two possibilities we examined extension at 20%–30% pupal development when most DCN axons are extending toward the medulla in wild-type flies. We find that inhibiting JNK activity results in an average of only 3.2 axons extending toward the medulla with 25% showing no extension (Figure 3D). These data are quantified in Figure 3G. Importantly, single-cell DCN FLP-out clones that express Bsk-DN also fail to extend their axons to the medulla; however, they appear to branch normally within the lobula and do not show obvious defects in guidance (Figure 3E–3E'), suggesting that JNK signaling affects DCN axon extension and likely not other aspects of DCN fate or development. To determine the full extent of the role of JNK signaling and whether it may act cell-autonomously in individual DCNs, we generated single DCN clones bearing a null allele of the Drosophila JNK *basket (bsk)* using the MARCM technique. MARCM mutant neurons are mutant from the moment of their birth and thus should completely lack JNK signaling from the onset of their differentiation. All *bsk* mutant clones examined lacked axons (Figure 3F and 3F'; Table 2) suggesting that JNK signaling is required to initiate DCN axon outgrowth. Not surprisingly, these neurons appeared unhealthy and stained relatively weakly for GFP, suggesting that they may be dying, perhaps due to the lack of connectivity and trophic support.

**Table 2:**
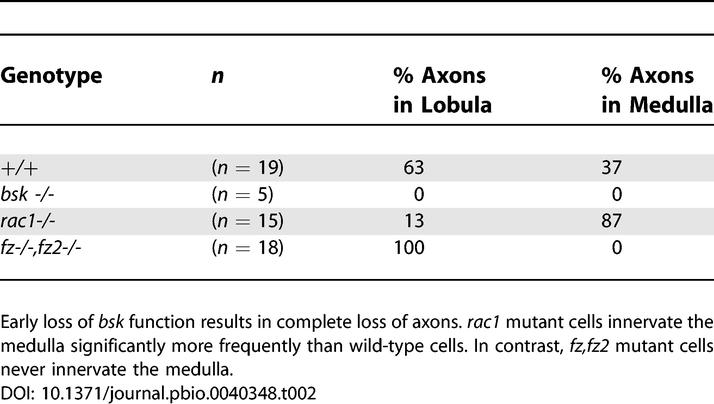
Results of MARCM Analysis for Wild-Type and *bsk, rac1,* and *fz,fz2* Mutant DCNs

To test if the JNK pathway is active in the DCNs, we evaluated the expression domains of the JNK pathway target gene *puckered (puc)* using a *puc-lacZ* reporter transgene [56]. We find that *puc-lacZ* is expressed in most, if not all neurons throughout the development of the adult brain (Figure S1A) including the DCNs (Figure S1A'), indicating that this pathway likely operates in the DCNs. Together, the results of these manipulations indicate that JNK signaling is necessary for DCN axon extension throughout brain development. Constitutively active JNK signaling in the DCNs is sufficient to override the normal retraction of a subset of DCN axons in an otherwise wild-type animal, suggesting the possibility that axonal retraction signal might operate through the attenuation of JNK activity.

### Rac1 GTPase in DCN Axon Outgrowth

**Figure 4:**
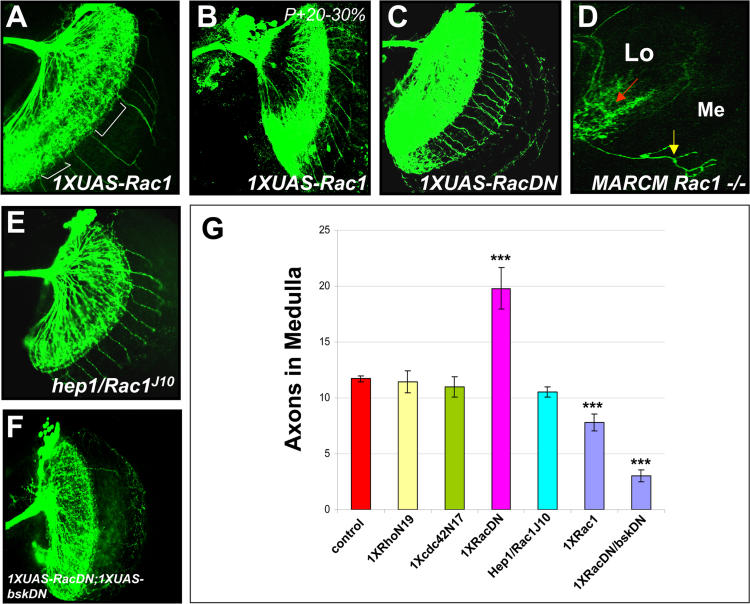
Rac1 Acts Upstream of JNK to Control DCN Axon Number (A) Confocal section through a *w; UAS::CD8GFP/UAS::Rac1; ato-Gal4-14a/+* adult brain, a reduction in the number of the axons is observed resulting in interruptions of the regular DCN axon pattern. (B) Confocal section through a *w; UAS::CD8GFP/UAS::Rac1; ato-Gal4-14a/+* pupal brain (P + 20%–30%). Fewer axons than normal are seen extending toward the medulla at this stage in these animals. (C) Confocal section through a *w; UAS::CD8GFP/+; ato-Gal4-14a/UAS::Rac1DN* adult brain. A marked increase in the number of the axons crossing the optic chiasm is observed. (D) Confocal section of a adult brain with a two-cell *Rac1* MARCM DCN clone (one in each hemisphere) obtained from *yw,hsFLP; UAS::CD8GFP/+; ato-Gal4-14a/+; Tub-gal80, FRT2A/Rac1^j11^, FRT2A* flies. Note that the contralateral axon crosses and branches in the medulla (yellow arrow). This is observed in 87% of all *Rac1* mutant cells compared to 37% of wild-type cells. Red arrow shows the ipsilateral dendrites of the DCN cell in the right hemisphere. (E) Confocal section through a *hep^1^/Y; UAS::CD8GFP/+; ato-Gal4-14a/Rac1^J10^* adult brain showing normal axon extension from the lobula to the medulla indicating that reduction of Rac1 levels rescues the loss of axon extension in *hep^1^* mutants. (F) Confocal section through a *UAS::bskDN/+; ato-Gal4-14a,lacZ/UAS::Rac1DN* adult brain: few axons are crossing the optic chiasm indicating a complete dominance of the *bsk* loss of function phenotype. (G) Quantification of the axonal extension phenotype for the genotypes shown in A, B, C, D, E, and F. A total of at least ten samples were evaluated for each genotype, axons were counted from each, and the average calculated (*** *p* < 0.001).

Our initial observations made with the dominant-negative form of *Rac1* [50] lead us to further investigate the roles of Rho GTPases in DCN axon extension. We expressed activated and dominant-negative forms of CDC42, RhoA, and Rac1, specifically in the DCNs. Neither activating (unpublished data) nor blocking RhoA or CDC42 had apparent effects on DCN axon extension. In contrast, activation of Rac1 inhibits DCN axon extension. Expression of a wild-type Rac1 transgene in DCNs decreased axon crossing from 11.7 to 7.8 with 92.8% of the samples showing less than nine axons crossing (Figure 4A). Expression of this transgene also resulted in a decreased number of DCN axons crossing the optic chiasm at 20%–30% pupal development (Figure 4B); albeit, not to the same extent as reduction of JNK signaling. Expression of activated Rac1 induces premature and severe retraction of DCN axons visible even at L3 (unpublished data). Conversely, blocking Rac1 activity using a dominant-negative Rac1 transgene results in an increased average number of axons crossing from the lobula to the medulla (11.7 to 19.8 with no samples with less than 17 axons crossing; Figure 4C). To determine whether Rac1 may act cell-autonomously in individual DCNs, we generated single *Rac1* mutant cell clones and counted the number of DCN axons crossing the optic chiasm. In contrast to wild-type animals, where only 38% of DCN axons innervate the medulla, 87% (13/15) of *Rac1* mutant axons do so (Figure 4D; Table 2). These data suggest that Rac1 and JNK have opposing effects on DCN axon extension and retraction.

To test if there is a genetic interaction between Rac1 and the JNK signaling pathway, we removed one copy of *Rac1* in the homozygous *hep^1^* background. We find that reduction of Rac1 expression levels fully rescues the decrease in axon extension in *hep^1^* mutants (Figure 4E). Since Rac1 and JNK act antagonistically, the simplest explanation is that activation of Rac1 represses JNK signaling. If so, *Rac1* would be expected to act upstream of, and antagonistically to *bsk.* Therefore, blocking both activities simultaneously should mimic the loss of Bsk and result in decreased axon extension. We therefore co-expressed both Rac1-DN and Bsk-DN and found that the *bsk* loss of function phenotype is epistatic to that of *Rac1* (an average of three axons crossing the optic chiasm with 80% of the samples showing less than three axons crossing; Figure 4F) suggesting that JNK signaling is downstream of Rac1. Quantification of these data is shown in Figure 4G.

### FGF Signaling Mediates DCN Axon Retraction

**Figure 5:**
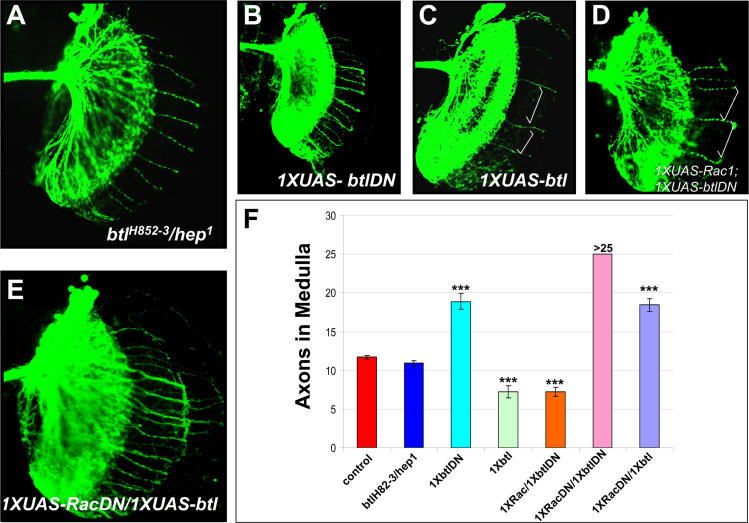
The FGFR Breathless Acts Upstream of Rac1 to Control DCN Axon Number (A) Confocal section through a *hep^1^/Y; UAS::CD8GFP/+; ato-Gal4-14a/btl^H852–3^* adult brain showing normal axon extension from the lobula to the medulla and therefore suppression of the *hep^1^* phenotype by reduction of *btl* levels. (B) Confocal section through a *w; UAS::CD8GFP/UAS::btlDN; ato-Gal4-14a/+* adult brain. A marked increase in the number of axons crossing the optic chiasm is observed. (C) Confocal section through a *w; UAS::CD8GFP/UAS::btl; ato-Gal4-14a/+* adult brain. A reduction in the number of axons is observed resulting in interruptions of the regular DCN axon pattern. (D) Confocal section through a *w; UAS::Rac1/UAS::btlDN; ato-Gal4-14a,UAS::lacZ/+* adult brain. A reduction in the number of axons is observed indicating the dominance of *Rac1* phenotype. (E) Confocal section through a *w; UAS::btl/+; ato-Gal4-14a,UAS::lacZ/UAS::RacDN* adult brain. A large increase in the number of axons crossing the optic chiasm is observed indicating the complete dominance of the *Rac1* phenotype. (F) Quantification of the axonal extension phenotypes for the genotypes shown in (A–E). A total of at least ten samples were evaluated for each genotype, axons were counted from each, and the average calculated (*** *p* < 0.001).

The function of Rac1 in regulating DCN axon retraction suggests that it may be the mediator of a retraction signal received by DCN axons during brain development. Clues to the nature of this putative retraction pathway came from the initial studies indicating that blocking the activity of the Drosophila FGFR *btl,* specifically in the DCNs, causes an increase in the number of axons extending from the lobula to the medulla (Table 1). To further address whether FGF signaling plays a role in DCN axon retraction, we evaluated possible genetic interactions between *hep^1^* and *btl.* Similar to Rac1, reduction of Btl levels rescues the axon extension defects observed in *hep^1^* mutants (Figure 5A). Next, we expressed dominant-negative [54] and wild-type Btl transgenes in the DCNs. We found that blocking Btl activity by expression of Btl-DN did not affect initial extension of DCN axons at 20%–30% pupal development (Figure S3A) but resulted in a large increase (from 11.7 to 18.9) in the number of DCN axons crossing toward the medulla in adult brains (Figure 5B); suggesting that FGF signaling is involved in inducing DCN axon retraction after the initial extension phase. Conversely, expression of wild-type Btl resulted in decreased numbers of axons crossing the optic chiasm (from 11.7 to 7.2) with the majority of samples (71.4%) showing less than seven axons crossing (Figure 5C).

As mentioned above, blocking the activity of the EGF receptor did not have any effect on the number of DCN axons extending between the lobula and the medulla. Thus, the effect we observe when FGF signaling is blocked is unlikely to be mediated by activation of the MAP kinase pathway common to both the EGFR and FGFR pathways. To confirm this, we misexpressed the MAP kinase pathway inhibitor Sprouty [57] in the DCN. No apparent effects on axon extension were observed (unpublished data); further suggesting that the FGF signaling-mediated retraction signal does not involve MAP kinase pathway members.

To confirm that the Drosophila FGF homolog, Bnl, is expressed during brain development, we examined its expression domains using a *bnl-lacZ* β-Gal reporter transgene [58]. We find that *bnl-lacZ* is expressed in dorso-ventral crescents of cells within the optic chiasm, as well as in the distal medulla during early (20%–30%) pupal development (Figure S1B). *bnl-lacZ* expression ceases late in pupal development and is no longer detectable in adult flies (unpublished data).

The similarity of the FGFR and *Rac1* phenotypes suggested that they might interact to control DCN axon retraction. If so, then the *loss and gain* of Rac1 function should be epistatic to the *gain and loss,* respectively, of Btl function. To determine if FGFR-dependent DCN retraction is mediated by Rac1 activation, we simultaneously blocked FGFR and overexpressed wild-type Rac1 in DCNs. We find that Rac1 gain-of-function phenotype (7.3 axons) is epistatic to FGFR loss-of-function (Figure 5D). Conversely, blocking Rac1 reverses the effects of overexpressing Btl and results in a Rac1 loss-of-function phenotype (Figure 5E). Finally, blocking both Btl and Rac1 together results in all DCN axons crossing the optic chiasm (Figure 5F), a phenotype similar to that observed with constitutive JNK signaling (Figure 3C). The phenotypes described above are quantified in Figure 5F.

In summary, our data suggest that DCN axons initially extend under the influence of an intrinsic JNK signal. During development, these axons encounter an FGF signal which results in Rac1 activation and the suppression of the extension-promoting JNK signal. This raises the question as to why some DCN axons do not retract and apparently stabilize along their paths. We therefore hypothesized the existence of a counter-retraction, or stabilization signal, required for the maintenance of the ~12 DCN axons that stably cross the optic chiasm.

### Wnt5 and Dsh Interact to Promote DCN Axon Extension

**Figure 6:**
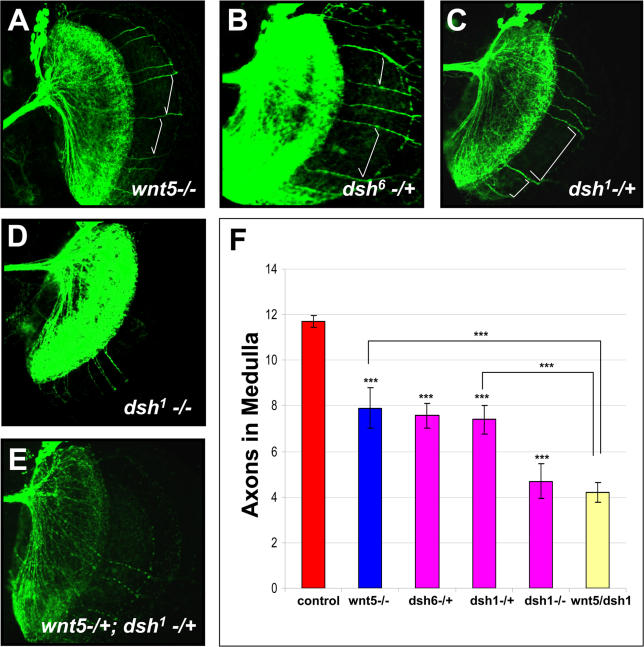
Wnt5 and Dsh Are Required for DCN Axon Stabilization (A) Confocal section through a *wnt5^400^; UAS::CD8GFP/+; ato-Gal4-14a/+* adult brain. A reduction in the number of the axons is observed. (B) Confocal section through a *dsh^6^/+; UAS::CD8GFP/+; ato-Gal4-14a/+* adult brain: a reduction in the number of the axons is observed resulting in interruptions of the regular DCN axon pattern. (C) Confocal section through a *dsh^1^/+; UAS::CD8GFP/+; ato-Gal4-14a/+* adult brain: a reduction in the number of the axons is observed resulting in interruptions of the regular DCN axon pattern. (D) Confocal section through a brain from *dsh^1^/Y; UAS::CD8GFP/+; ato-Gal4-14a/+* animal showing a more severe reduction in the number of axons crossing the optic chiasm. (E) Confocal section through a double heterozygous *wnt5/dsh^1^; ato-Gal4-14a,UAS::,lacZ/+* adult brain showing a more severe phenotype in the number of the axons reflecting a synergistic interaction between *wnt5* and *dsh.* (F) Quantification of the axonal extension phenotype for the genotypes shown in (A–E). A total of at least ten samples were evaluated for each genotype, axons were counted from each, and the average calculated (*** *p* < 0.001).

Based on our observation that blocking Fz2 resulted in decreased numbers of DCN axons in the medulla, we reasoned that Fz2 could be a receptor for the putative stabilization signal. As Fz2 and Fz are partially redundant receptors for the canonical Wnt signaling pathway [59], we investigated where the canonical Wnt ligand wingless (Wg) is expressed in the brain during pupation. However, we did not detect Wg expression in the pupal optic lobes (unpublished data), suggesting that Wg is unlikely to be involved in regulating DCN axon extension. We therefore examined the expression of Wnt5, which has been shown to be involved in axon repulsion and fasciculation in the embryonic CNS [26,60]. Anti-Wnt5 staining revealed widely distributed Wnt5 expression domains beginning at PF (Figure S1C) and lasting throughout pupal development and into adult life (Figure S1D). Wnt5 is strongly expressed in the distal medulla and is also present on axonal bundles crossing the second optic chiasm (Figure S1D, arrows). We evaluated the number of DCN axons crossing to the medulla in *wnt5* mutant flies. We find that the number of DCN axons crossing the optic chiasm is reduced from 11.7 to 7.9 in the absence of *wnt5* (Figure 6A), suggesting that it may play a role in stabilizing DCN axons.

Next, we tested the requirement of the Wnt signaling adaptor protein Dsh. In animals heterozygous for *dsh^6^,* a null allele of *dsh,* the average number of DCN axons crossing between the lobula and the medulla is reduced from 11.7 to 7.6 with 78.5% showing less than eight axons crossing (Figure 6B). Signaling through Dsh is mediated by one of two domains [61]. Signaling via the DIX (Disheveled and Axin) domain is thought to result in the activation of Armadillo/β-Catenin. DEP (Disheveled, Egl-10, Pleckstrin) domain-dependent signaling results in activation of the JNK signaling pathway by regulation of Rho family GTPase proteins during, for example, convergent extension movements in vertebrates [62,63]. To uncover which of these two pathways is required for DCN axon extension we used the *dsh^1^* mutant that is deficient only in the activity of the DEP domain [64]. Indeed, in brains from *dsh^1^* heterozygous animals the number of extending axons was reduced from 11.7 to 7.4 (Figure 6C). In flies homozygous for the *dsh^1^* allele the average number of axons crossing was further reduced to 4.7, with all the samples having less than six axons crossing (Figure 6D). In contrast, the DCN-specific expression of Axin, a physiological inhibitor of the Wnt canonical pathway, did not affect the extension of DCN axons. Similarly, expression of a constitutively active form of the fly β-Catenin Armadillo also had no apparent effect on DCN extension (unpublished data). Finally, we tested if Wnt5 and Dsh interact synergistically. To this end, we generated *wnt5, dsh^1^* trans-heterozygous animals. These flies show the same phenotype as flies homozygous for *dsh^1^* (Figure 6E), suggesting that Wnt5 signals through the Dsh DEP domain. The data above are quantified in Figure 6F.

To determine if *dsh* is expressed at times and places suggested by its genetic requirement in DCN axon outgrowth, we examined the distribution of Dsh protein during brain development. We find that Dsh protein is ubiquitously expressed during brain development (Figure S2A and S2B). High expression of Dsh is detected in the distal ends of DCN axons at about 15% PF shortly before they extend across the optic chiasm toward the medulla (Figure S2A, arrows). In general, we observe higher levels of Dsh in the neuropil than in cell bodies (Figure S2B).

In summary, our data indicate that the stabilization of DCN axons is dependent on the Dsh protein acting non-canonically via its DEP domain. Importantly, the axons that do cross in *dsh* mutant brains do so along the correct paths. This suggests that, like JNK signaling, Wnt signaling regulates extension, but not guidance, of the DCN axons.

### Wnt5 Signals to the DCNs via the Frizzled Receptors

**Figure 7:**
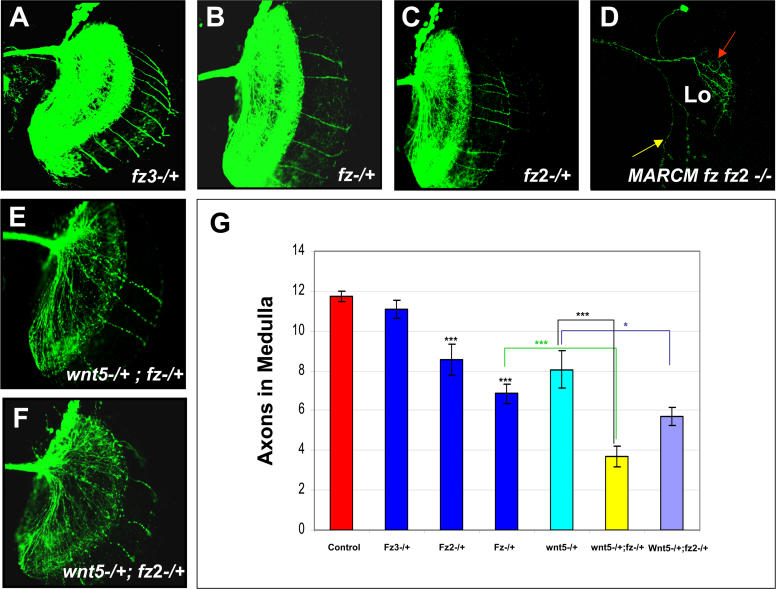
Wnt5 Acts through Fz Receptors to Control DCN Axon Extension (A) Confocal section through a *fz3/Y; UAS::CD8GFP/+; ato-Gal4-14a/+* adult brain. No significant effect is observed. (B) Confocal section through a *w; UAS::CD8GFP/+; ato-Gal4-14a/fz* adult brain. A small, but significant reduction in the number of the axons is observed. (C) Confocal section through a *w; UAS::CD8GFP/+; ato-Gal4-14a/fz2* adult brain. A significant reduction in the number of the axons is observed. (D) Confocal section of a adult brain with a two-cell *Fz, Fz2,* MARCM DCN clone (one in each hemisphere) obtained from *yw,hsFLP; UAS::CD8GFP/+; ato-Gal4-14a/+; Tub-gal80, FRT2A/Fz ^h51^ Fz2^C1^, FRT2A* flies. The contralateral axons fail to extend to the medulla and instead innervate the lobula (yellow arrow). This is observed in 100% of the mutant clones examined. Red arrow shows the ipsilateral dendrites of the single DCN cell in the right hemisphere. (E) Confocal section through a *wnt5/+; ato-Gal4-14a,UAS::lacZ/fz* adult brain: a synergistic reduction in the number of axons compared to *wnt5/+* and *fz/+* animals is observed. (F) Confocal section through a *wnt5/+; ato-Gal4-14a,lacZ/fz2* showing a small, but significant difference to *wnt5/+* animals. (G) Quantification of the axonal extension phenotype. A total of at least ten samples were evaluated for each genotype, axons were counted from each, and the average calculated (*** *p* < 0.001; * *p* < 0.03).

Wnt signaling to Dsh requires the Fz receptors [65]. To examine if the effect of Wnt5 on DCN axon extension is also mediated by Fz receptors, we counted the number of DCN axons crossing the optic chiasm in *fz, fz2,* and *fz3* mutants. There was no significant change in the number of axons crossing in the brain of *fz3* homozygous animals (Figure 7A). In contrast, in brains heterozygous for *fz* and *fz2,* the number of the axons crossing was reduced from 11.7 to 6.6 *(fz)* and 6.9 *(fz2),* with 71% and 85.7%, respectively, showing less than eight axons crossing (Figure 7B and 7C). These data suggest that DCN axons respond to Wnt5 using the Fz and Fz2 receptors, but not Fz3. To determine whether the Fz receptors act cell-autonomously in individual DCNs, we generated single-cell clones doubly mutant for *fz* and *fz2* and counted the number of DCN axons crossing the optic chiasm. In contrast to wild-type cells, where 37% of all DCN axons cross, none (0/18) of the *fz, fz2* mutant axons reach the medulla (Figure 7D; Table 2). To test whether *wnt5, fz,* and *fz2* genetically interact in DCNs, we examined flies trans-heterozygous for *wnt5* and both receptors. We find that flies heterozygous for both *wnt5* and *fz* mutations show a strong synergistic loss of DCN axons (11.7 to 3.7; Figure 7E) and in fact have a phenotype very similar to that of flies homozygous for *dsh^1^.* Flies doubly heterozygous for *wnt5* and *fz2* (Figure 7F) also show a significant decrease in DCN axons (5.7), compared with either *wnt5* (~8) or *fz2* (8.5) mutants. The data above are quantified in Figure 7G. Our data indicate that the genetic interaction between *wnt5* and *fz* is stronger than the interaction between *wnt5* and *fz2.*

Examination of the expression domains of Fz and Fz2 in the developing brain supports the possibility that they play roles in stabilizing DCN axons. Both Fz and Fz2 are widely expressed in the developing adult brain neuropil. In addition, Fz is expressed at higher levels in DCN cell bodies (Figure S2C and S2D).

**Figure 8:**
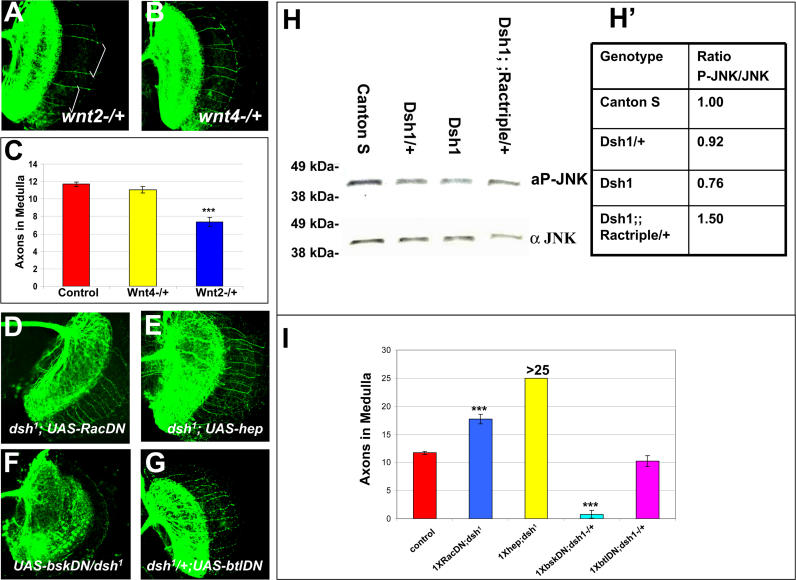
Dsh and Rac Modulate JNK Activation (A) Confocal section through a *w; UAS::CD8GFP/wnt2; ato-Gal4-14a/+* adult brain. A reduction in the number of the axons is observed. (B) Confocal section through a *w; UAS::CD8GFP/wnt4; ato-Gal4-14a/+* adult brain. No reduction in the number of axons is observed. C) Quantification of the *wnt2* and *wnt4* effects on DCN axon extension. A total of at least ten samples were evaluated for each genotype, axons were counted from each, and the average calculated (*** *p* < 0.001). (D) Confocal section through a *dsh^1^/Y; ato-Gal4-14a, UAS::lacZ/UAS::RacDN* adult brain. An increase in the number of the axons crossing the optic chiasm is observed indicating a complete dominance of the Rac1 phenotype. (E) Confocal section through a *dsh1/Y; UAS::hep/+; ato-Gal4-14a,lacZ/+* adult brain. A large increase in the axons crossing the optic chiasm is observed indicating a complete dominance of the *hep* gain of function phenotype. (F) Confocal section through a *UAS::bskDN/dsh1; ato-Gal4-14a,UAS::lacZ/+* adult brain. Almost no axons are crossing the optic chiasm. (G) Confocal section through a *dsh1/+; UAS:: btlDN /+; ato-Gal4-14a,lacZ/+* adult brain. No significant difference in the number of the axons crossing the optic chiasm compared with control flies. However, there is an increase in variability as indicated by the larger error bar in (I) compared to control flies. (H) Heads from Canton S, *dsh^1^/+, dsh^1^/dsh^1^, dsh^1^/dsh^1^;; RacJ11,Rac2* ^Δ^ *FRT80,Mtl* ^Δ^ adults animals were lysed, and whole-head lysates were subjected to SDS-PAGE. Phosphorylated and total JNK levels were examined by immunoblot analysis with anti-P-JNK (upper panel) and total JNK antibodies (bottom panel), respectively. (H') JNK activity is calculated as the relative amount of P-JNK to total JNK. Loss of Dsh function leads to a 25% decrease in JNK activity, which is rescued to 150% of wild-type levels by reduction of Rac levels showing mutually antagonistic effects of Dsh and Rac on JNK activity. (I) Quantification of the axonal extension phenotype. A total of at least ten samples were evaluated for each genotype, axons were counted from each, and the average calculated (*** *p* < 0.001).

The observation that the *wnt5* null phenotype can be enhanced by reduction of Fz, Fz2, or Dsh suggests that another Wnt may be partially compensating for the loss of Wnt5. To test this possibility, we examined flies heterozygous for either *wnt2* or *wnt4.* We find that *wnt2* heterozygotes display a reduction of DCN axon crossing from 11.7 to 7.3, whereas no phenotype was observed for *wnt4* (Figure 8A–8C). Thus, *wnt2* and *wnt5* may act together to stabilize the subset of DCN axons that do not retract during development. In summary, these results support the model that Wnt signaling via the Fz receptors transmits a non-canonical signal through Dsh resulting in the stabilization of a subset of DCN axons.

### A Balance of Wnt and FGFR-Rac Signaling Regulates JNK Function in DCN Axon Extension

Since our data support the hypothesis that the regulation of JNK by Rac1 modulates DCN axon extension, we sought to determine how Wnt signaling might interact with Rac1 and JNK. The opposite phenotypes of *dsh* and *Rac1* loss-of-function suggest that they might act antagonistically. To determine if Rac1 is acting upstream of, downstream of, or in parallel to Dsh in DCN axon extension, we expressed dominant-negative Rac1 in *dsh^1^* mutant flies. If Rac1 acts upstream of Dsh, we expect the *dsh^1^* phenotype (i.e., decreased numbers of axons crossing the optic chiasm). If Rac1 acts downstream of Dsh, we would expect the *Rac1* mutant phenotype (i.e., increased number of axons crossing). If they act in parallel, we expect an intermediate, relatively normal phenotype. We observe increased numbers of axon crossing (Figure 8D), suggesting that Rac1 acts downstream of Dsh during DCN axon extension and that Dsh may repress Rac1.

Next, we tested if Dsh control of DCN axon extension is mediated by the JNK signaling pathway acting downstream of Wnt signaling, as the similarity of their phenotypes suggests. If this were the case, we expected that activating JNK signaling should suppress the reduction in Dsh levels. Conversely, reducing both should show a synergistic effect. We therefore expressed the JNKK *hep* in *dsh^1^* heterozygous flies and found that the *hep* gain-of-function is epistatic to *dsh* loss-of-function (Figure 8E). Furthermore, reducing JNK activity by one copy of BSK-DN in *dsh^1^* mutant animals results in a synergistic reduction of extension to an average of 0.8 axons with 60% showing no axons crossing and no samples with more than three axons (Figure 8F). Quantification of these data is shown in Figure 8I. In summary, the results of our genetic analyses suggest that Wnt signaling via Dsh enhances JNK activity through the suppression of Rac1.

Dsh appears to promote JNK signaling and to be expressed in DCN axons prior to their extension toward the medulla early in pupal development (Figure S2A). Since JNK signaling is required for this initial extension, it may be that Dsh also plays a role in the early extension of DCN axons. To test this possibility, we examined DCN axon extension at 30% pupal development in *dsh^1^* mutant brains. In wild-type pupae, essentially all (~40) DCN axons extend toward the medulla (Figure 1F and 1G). In contrast, in *dsh^1^* mutant pupae, we observe a strong reduction in the number of DCN axons crossing the optic chiasm between the lobula and the medulla (Figure S3B).

Although the genetic data indicate that Dsh- and Rac-mediated signaling have sensitive and antagonistic effects on the JNK pathway, they do not establish whether the Dsh-Rac interaction modulates JNK's intrinsic activity. To test this, we evaluated the amount of phosphorylated JNK relative to total JNK levels in fly brains by Western blot analysis using phospho-JNK (P-JNK) and pan-JNK specific antibodies. We then determined if Dsh is indeed required for increased levels of JNK phosphorylation. We find that *Dsh^1^* mutant brains (Figure 8H, lane 3) show a 25% reduction in P-JNK (Figure 8H', *n* = 3) consistent with a stimulatory role for Dsh on JNK signaling. The reduction caused by loss of *Dsh* function is reversed (Figure 8H, lane 4), to 150% of wild-type levels (Figure 8H'), when the amount of Rac is reduced by half, consistent with a negative effect of Rac on JNK signaling downstream of Dsh. These data support the conclusion that Dsh and Rac interact to regulate JNK signaling by modulating the phosphorylated active pool of JNK.

Taken together, our data suggest that during brain development DCN axons extend under the influence of JNK signaling. A non-canonical Wnt signal acting via Fz and Dsh ensures that JNK signaling remains active by attenuating Rac activity. In contrast, activation of the FGFR activates Rac1 and suppresses JNK signaling. These data support a model whereby the balance of the Wnt and FGF signals is responsible for determining the number of DCN axons that stably cross the optic chiasm. To test this model, we reduced FGFR levels, using the dominant-negative *btl* transgene, in *dsh^1^* heterozygous flies. We found that simultaneous reduction of FGF and Wnt signaling restored the number of axons crossing the optic chiasm to almost wild-type levels (10.2, with 33% of the samples indistinguishable from wild-type; Figure 8G and 8I), suggesting that the two signals in parallel, to control the patterning of DCN axon connectivity.

## Discussion

In this study we investigated how axon extension and retraction contribute to the patterning of neuronal connections using the visual system of the Drosophila brain as a model. The Drosophila brain is well suited for these analyses in that it is both highly complex and amenable to genetic analysis. In addition, an increasing number of well-defined and specifically marked neuronal populations allow the in vivo visualization of complex events during brain development. One such population, the DCNs, represents an attractive system in which to investigate these issues due to their stereotypical and readily detectable axonal projection pattern. This regular pattern allows quantitative assessment of the effects of specific mutations on an entire group of related neurons, at the same time providing single axon resolution. We find that the development of the adult pattern of DCN connections does not require neuronal activity, suggesting that it is specified by an intrinsic DCN genetic program and extracellular cues.

Our work reveals a regulatory network composed of at least two signaling pathways, Wnt and FGF, which feed into common downstream effectors, Rac and JNK, which serve to regulate the dynamic behavior of axons during development. Members of these pathways localize to the DCNs or their target regions during the DCN axon remodeling stages, suggesting that the genetic results reflect their roles there. Axons extending to the medulla in the mutant backgrounds we examined appear to project along normal paths. Thus, the Wnt-FGF regulatory network described here appears to mediate axonal extension and retraction, but not axon guidance, suggesting that these aspects of neuronal connectivity can be regulated independently.

**Figure 9:**
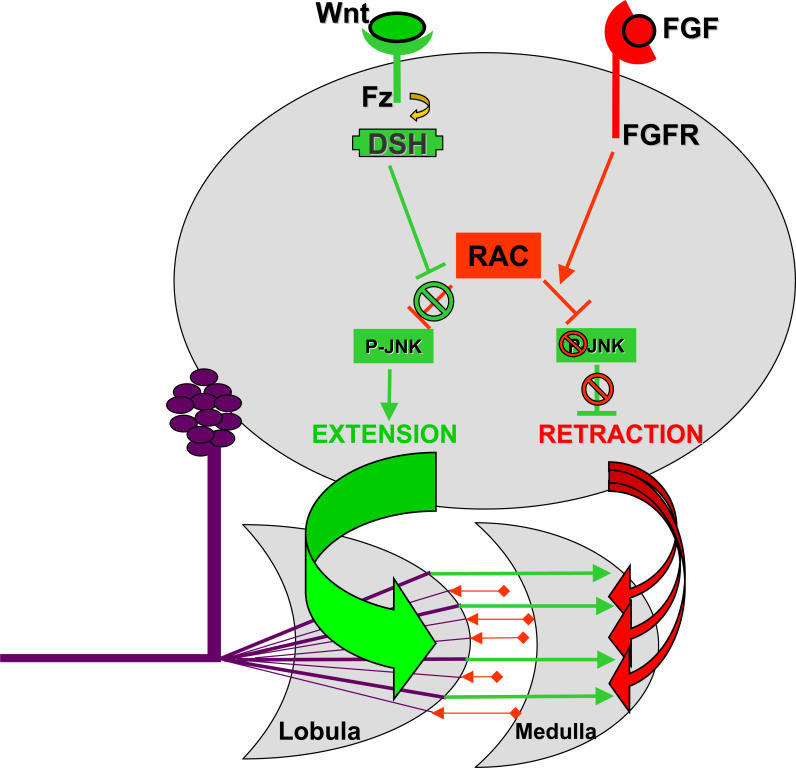
Schematic Model Representing the Interaction and Integration of the Signals Controlling the DCN Axon Extension Our data suggest the following model of DCN axon extension and retraction. DCN axons extend due to active JNK signal. These axons encounter Wnt5 and probably Wnt2 as well, resulting in activation of Disheveled. Disheveled, via its DEP domain, has a negative effect on the activity of the Rac GTPase, thus keeping JNK signaling active. After DCN axons cross the second optic chiasm they encounter a spatially regulated FGF/Branchless signal that activates the FGFR/Breathless pathway. Breathless in turn activates Rac, which inhibits JNK signaling in a subset of axons. These axons then retract back toward the lobula. The wide expression of the different components of these pathways and the modulation of JNK phosphorylation by Dsh and Rac in whole-head extracts strongly suggests that this model may apply to many neuronal types.

Together, the data suggest a model (Figure 9) in which extending axons encounter competing signals that cause them to retract or to continue to extend and stabilize in their specific paths. Analysis of single-cell mutant clones in otherwise wild-type backgrounds indicates that JNK signaling is required cell-autonomously for initial axon outgrowth. Inhibition of JNK signaling during later phases of axonal extension using a dominant-negative transgene prevents axons from extending toward their targets. JNK signaling therefore appears to be required at these two, and perhaps all stages of DCN axon outgrowth.

Signaling by Wnt proteins via Fz receptors and Dsh, provides an important stabilization signal, likely by mediating the continued activation of JNK. Wnts have recently been shown to be involved in the establishment of neuronal polarity that orients anterior-posterior axon outgrowth [67,68]. Initial DCN axon outgrowth occurs normally in *wnt* mutants suggesting that either other Wnt ligands or non-Wnt pathways are involved. Single-cell MARCM analyses indicate that the *fz/fz2* receptors, which we find to interact genetically with *wnt5,* are required cell-autonomously in the DCNs.

Wnt5 acts to repulse a subset of embryonic CNS axons, apparently independently of Dsh and the Fz receptors, via the RYK tyrosine kinase receptor family member Drl [26]. One way to reconcile those findings with these presented here is to propose that the specific receptor interacting with Wnt5 determines axonal behavior. Support for this hypothesis comes from the recent observations that purified Wnt5a protein can either activate or inhibit β-Catenin signaling, depending on which of the two receptors it interacts with [69]. Furthermore, the interaction of Wnt proteins with potentially alternative receptors need not be mutually exclusive, as suggested by a study of Wnt interactions with Fz and RYK in mammalian tissue culture cells [70]. Our data indicate that Wnt5 binding to the Fz receptors likely stabilizes axons, whereas Wnt5 signaling through Drl is apparently interpreted by embryonic CNS axons as a repulsive signal. Consistent with this, we observe that ectopic expression of Drl in DCNs results in a reduction of the number of DCN axons crossing the optic chiasm (from 11.7 to ~8; unpublished data). We do not, however, observe Drl expression in DCNs, making it unlikely that Drl plays a role in regulating the DCN connectivity pattern.

Conversely, activation of FGFR signaling results in axonal retraction, apparently through the inhibition of JNK signaling. The Rac1 GTPase and JNK appear to act to integrate these pathways: the Wnt and FGFR extracellular cues modulate Rac1 activity, which in turn attenuates JNK signaling, and therefore the balance between axon extension and retraction. Thus, Rac1 transforms complex signaling events into a deterministic binary decision. This decision is likely to be made autonomously in each DCN cell, since we observe that Rac1 mutant DCN single-cell clones cross the optic chiasm at significantly higher rates than controls. A recent study shows that in contrast to what we observe in the Drosophila DCNs, Wnts can signal through Rac to increase JNK signaling to promote dendritic arborization in mammals [71]. The differences between these two results of Wnt signaling through Rac may be accounted for by observations that Rac can have opposite effects on outgrowth in axons versus dendrites [72].

Remodeling of neuronal connections by changes in axonal behavior may result from either retraction or degeneration of axonal shafts and branches. Axonal degeneration, including the blebbing and fragmentation of the axonal shaft, has been shown to occur in both fly and vertebrate axonal remodeling [41,42]. Alternatively, axons may retract from one target site to another, a process which has been suggested to involve the Rac GTPase [66]. During the remodeling of DCN axonal connections, it appears that the axons retract rather than degenerate. This is supported by the lack of DCN axonal fragments in the medulla during the remodeling phase, as well as the requirement of Rac activity for the remodeling process.

The development of the adult DCN pattern did not require neuronal activity, suggesting that it is part of a hard-wired genetic program running in the DCNs either as individual cells or as a group. In order to generate a more complete picture of how this genetic program establishes a connectivity map, it will be important to determine the mechanism that specifies the identity of those axons stabilized by Wnt signaling. Are they predetermined by programmed differences between the individual DCNs? Alternatively, are they stochastically specified with competitive interactions resolving the numbers of axons crossing the optic chiasm? Finally, how is the regular spacing between DCN axons determined? Although single-cell mutant analysis suggests that whether or not a given DCN axon retracts or stabilizes is determined in part by cell-autonomous machinery, our data do not exclude a requirement for interactions between individual DCN cells. Thus, while many of the genes examined in this study, e.g., *fz, fz2, bsk,* and *rac1,* apparently act cell-autonomously, it remains possible that DCN axons engage in competitive interactions, or that the retraction or extension of certain axons influences whether other axons retract or continue to extend. Addressing these issues will enable us to understand the rules governing the generation of the formidable complexity of neuronal connectivity which underlies animal behavior.

## Materials and Methods

### Drosophila strains and genetics.

All stocks were raised on standard fly food and the crosses were performed in controlled 25 °C incubators. The *Tb* and *Hu* markers on the *TM6B* balancer chromosome and the *L* marker and *CyO* chromosome were used to distinguish mutant animals from their heterozygous siblings. The following stocks, referenced throughout the manuscript, were used: *atoGal4–10, atoGal4-14A, wnt2^0^, wnt4^EMS23^, wnt5^400^, dsh^1^, dsh^6^, dfz3^G10^, fz2^C1^, fz2^E4^, dfz^KD^, DrlRed2, UAS::Rac1^V12^, UAS::Rac1^N17^, UAS::RhoA^V14^, UAS::RhoA^N19.22^, UAS::Cdc42^N17.3^, UAS::Cdc42^V12.8^, UAS::shi^K44A^4–1, UAS::shi^K44A^3–7, UAS::shi^K44A^3–10, UAS::dORK^ΔC-1^, UAS::dORK^ΔC-2^, UAS-spry, UAS::DER^DN^, UAS::hep^CA^, UAS::bsk^DN^, UAS::dAxin, UAS::arm^act^, UAS::fz2GPI, UAS::bt1^DN^, UAS::btl, puc-lacZ, bnl-lacZ, hs-FLP, FRT40, tubGAL80/bsk^2^, FRT40; ato-Gal4-14a,UAS::lacZ/+, yw,hsFLP; UAS::CD8GFP/+; ato-Gal4–10/+; Tub-gal80, FRT2A/Rac1^j11^,* FRT2A.

### Generation of MARCM clones.

Generation of DCN MARCM clones: 36-h to 72-h-old larvae in a vial of standard fly food were heat shocked at 37 °C for 1 h. Expression of hs-FLP transgene was induced and hence mitotic recombination. After clone induction, the animals were kept at 25 °C. Brains were dissected at adult stage and subjected to immunohistochemistry.

### Expression analysis.

Immunohistochemistry was performed as described [19], except for Wnt5. Briefly, L3, pupal, or adult brains were dissected in PBS (phosphate buffered saline) and fixed with 4% formaldehyde in 1X PBT (1X PBS + 0.3% Triton X-100) for 20 min. Fixed brains were incubated overnight in 1X PAXDG Buffer (PBT, 5% normal goat serum, 1% bovine serum albumin, 0.1% deoxycholate, and 1% Triton X-100) with either anti-Dsh (T. Uemura), anti-β-galactosidase (Promega, Madison, Wisconsin, United States), anti-Elav (DSHB), anti-fz 1C11 (DSHB), anti-Fz2 12CA7 (DSHB), and anti-GFP (Molecular Probes, Eugene, Oregon, United States). This incubation was followed by several washes in PBT (1 h) and a final incubation with the appropriate fluorescent secondary antibodies (*Alexa* 488, 555, or 647, Molecular Probes, 1:500, 2 h). Affinity-purified anti-Wnt5 antibody [60] was used to stain unfixed brains in PBS for 40 min. The stained brains were washed three times in PBS and subsequently fixed in 4% formaldehyde prior to incubation with a fluorescent secondary antibody. Visualization was by confocal microscopy (BioRad 1024, Hercules, California, United States and Leica DM-RXA, Wetzlar, Germany)

### Western blotting.

For determination of P-JNK levels, flies were raised at 25 °C. Adult flies of the appropriate genotype were collected and decapitated. Four heads were homogenized in 40 μl of sample buffer (0.1 M Tris-HCl [pH6.8], 4% SDS, 20% Glycerol, 5% mercaptoethanol) on ice and immediately boiled at 96 °C. 15 μl of the samples were run per lane on a 4%–15% polyacrylamide Tris-HCl Precast gel (Bio-Rad) and electrophoretically transferred to Hybond nitrocellulose membrane (Amersham Biosciences, Little Chalfont, United Kingdom). Blots were probed with anti-P-JNK antibody (Cell Signaling, Danvers, Massachusetts, United States), developed with chemiluminescence reagents (ECL, Amersham Biosciences), and exposed to Hyperfilm ECL (Amersham Biosciences). P-JNK levels were normalized to total JNK levels detected using anti-JNK antibodies (Cell Signaling). Blots were repeated at least three times and quantified using the Scion Imaging software.

### Statistical analysis.

Genotypes were compared by one-tailed *t*-tests, using the data analysis software *STATSTICA* (StatSoft Incorporated, 2001, version 6.0).

## Supporting Information

Figure S1 JNK, FGF, and Wnt5 Are Expressed during Adult Brain Development (A) A late pupal brain from a *w; UAS::CD8GFP; ato-Gal4–14/puc-lacZ* transgenic fly stained for β-Gal (red) and GFP (green). Most if not all neurons show β-Gal expression. (A') Magnification of confocal section through the brain of *w; UAS::CD8GFP; ato-Gal4–14/puc-lacZ* animal stained for GFP (green) and β-Gal (red) showing colocalization of the β*-*Gal and GFP signals in the DCN (arrows).
(B) A pupal brain from a *bnl-lacZ* fly stained for β-Gal protein. β-Gal is expressed in discrete cells along the dorso-ventral axis (arrows) within the optic chiasm. Clusters of β-Gal-expressing cells are also observed within the medulla.
(C) An early pupal brain (P + 20%) from a *w; UAS::CD8GFP/+; ato-Gal4-14a/+* fly stained with α-GFP (green) and α-Wnt5 (red) antisera. Wnt5 is highly expressed in the central brain and in the medulla, target of the DCN axons (green) shortly before they begin to extend.
(D) A late pupal brain stained with the Wnt5 antibody. Wnt5 is widely expressed in the central brain and the optic lobes. High expression is observed in the medulla. In the optic chiasm between the lobula and the medulla Wnt5 is expressed in alternating stripes of high and low levels along the dorso-ventral axis (arrows).
(9.6 MB PDF)

Figure S2 Dsh and the Fz Receptors Are Expressed during Adult Brain Development (A) Pupal brain from a *w; UAS::CD8GFP/+; ato-Gal4-14a/+* animal (P + 15%) stained with α-GFP (green) and α-Dsh (red). High expression of Dsh is detected in the growth cones of the DCN axons (arrow), as well as widely in the developing neuropils.
(B) *w; UAS::CD8GFP/+; ato-Gal4-14a/+* late pupal brain stained with α-GFP (green), the nuclear pan-neuronal marker α-Elav (red) and α-Dsh (blue) confirming that Dsh is highly expressed in the neuropil.
(C) Pupal brain from a *w; UAS::CD8GFP/+; ato-Gal4-14a/+* animal (P + 30%) stained with α-Fz2. High expression of Fz2 is detected in the optic lobes.
(D) A pupal brain from a *w; UAS::CD8GFP; ato-Gal4–14* transgenic fly stained for GFP (green) and Fz (red). Most if not all DCNs show Fz expression. Colocalization of GFP (green) and Fz (red) in the DCN (arrows).
(9.1 MB PDF)

Figure S3 Effect of FGF and Wnt Signaling on DCN Axons Crossing during Early Brain Development (A) Confocal section through a *w; UAS::CD8GFP/UAS::btlDN; ato-Gal4-14a/+* pupal brain (P + 20%–30%). No effect on the initial extension of DCN axons was observed. (B) Confocal section through a *dsh^1^/Y; UAS::CD8GFP/+; ato-Gal4-14a/+* pupal brain (P + 20%–30%) showing a strong reduction in the number of axons crossing the optic chiasm.
(4.6 MB PDF)

## Abbreviations

β-Gal: β-Galactosidase
Bnl: Branchless
Btl: Breathless
DCN: dorsal cluster neuron
DEP: Disheveled Egl-10 Pleckstrin
Dsh: Disheveled
EGF: epidermal growth factor
FGF: fibroblast growth factor
FGFR: fibroblast growth factor receptor
FLP: Flipase-mediated recombination
fz: Frizzled
GFP: green fluorescent protein
JNK: Jun N-terminal kinase
L3: third instar larval
MARCM: mutant analysis using a repressible cell marker
PF: puparium formation
P-JNK: Phospho-JNK
