# Correction: A Signaling Network for Patterning of Neuronal Connectivity in the Drosophila Brain

**DOI:** 10.1371/journal.pbio.0040432

**Published:** 2006-12-12

**Authors:** Mohammed Srahna, Maarten Leyssen, Ching Man Choi, Lee Fradkin, Jasprina Noordermeer, Bassem Hassan

In *PLoS Biology*, volume 4, issue 11: DOI: 10.1371/journal.pbio.0040348

The author contributions were listed incorrectly. The correct author contributions are as follows: BAH conceived and designed the experiments. MS, ML, JNN, LGF, CMC, and BAH performed the experiments and analyzed the data. MS, LGF, JNN, and BAH wrote the paper.

