# Supplementary material for: A Signaling Network for Patterning of Neuronal Connectivity in the Drosophila Brain
Source: PLoS Biol. 2006 Oct 10;4(11):e348. doi: 10.1371/journal.pbio.0040348 (PMC1592317; doi:10.1371/journal.pbio.0040348)
Supplement: Supplementary file 1 [file pbio.0040348.sg001.pdf]

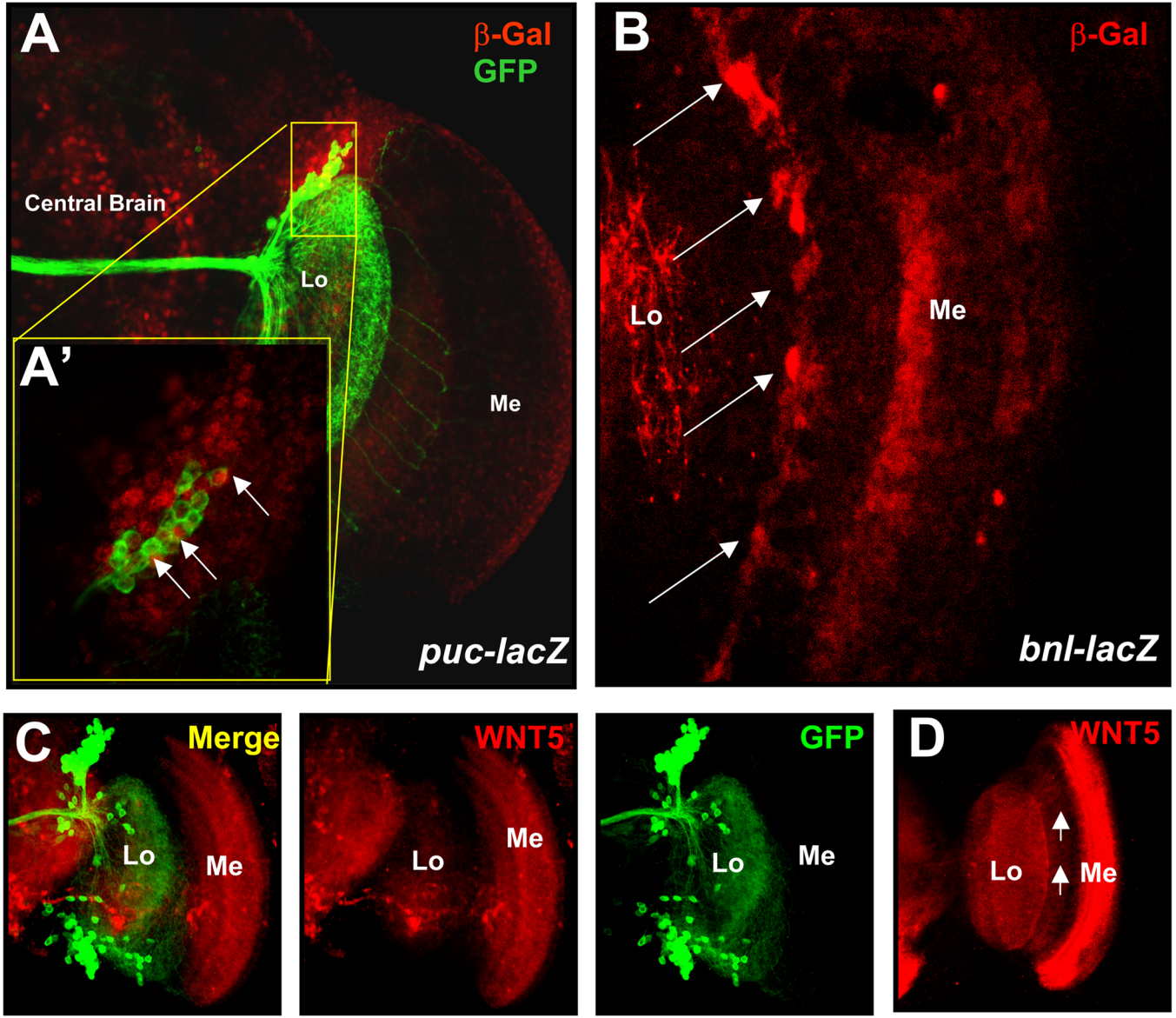
A
β-Gal
GFP
Central Brain
Lo
Me
A'
puc-lacZ
B
β-Gal
Lo
Me
bnl-lacZ
C
Merge
Lo
Me
WNT5
Lo
Me
GFP
Lo
Me
D
WNT5
Lo
Me
