# Supplementary material for: A Signaling Network for Patterning of Neuronal Connectivity in the Drosophila Brain
Source: PLoS Biol. 2006 Oct 10;4(11):e348. doi: 10.1371/journal.pbio.0040348 (PMC1592317; doi:10.1371/journal.pbio.0040348)
Supplement: Supplementary file 2 [file pbio.0040348.sg002.pdf]

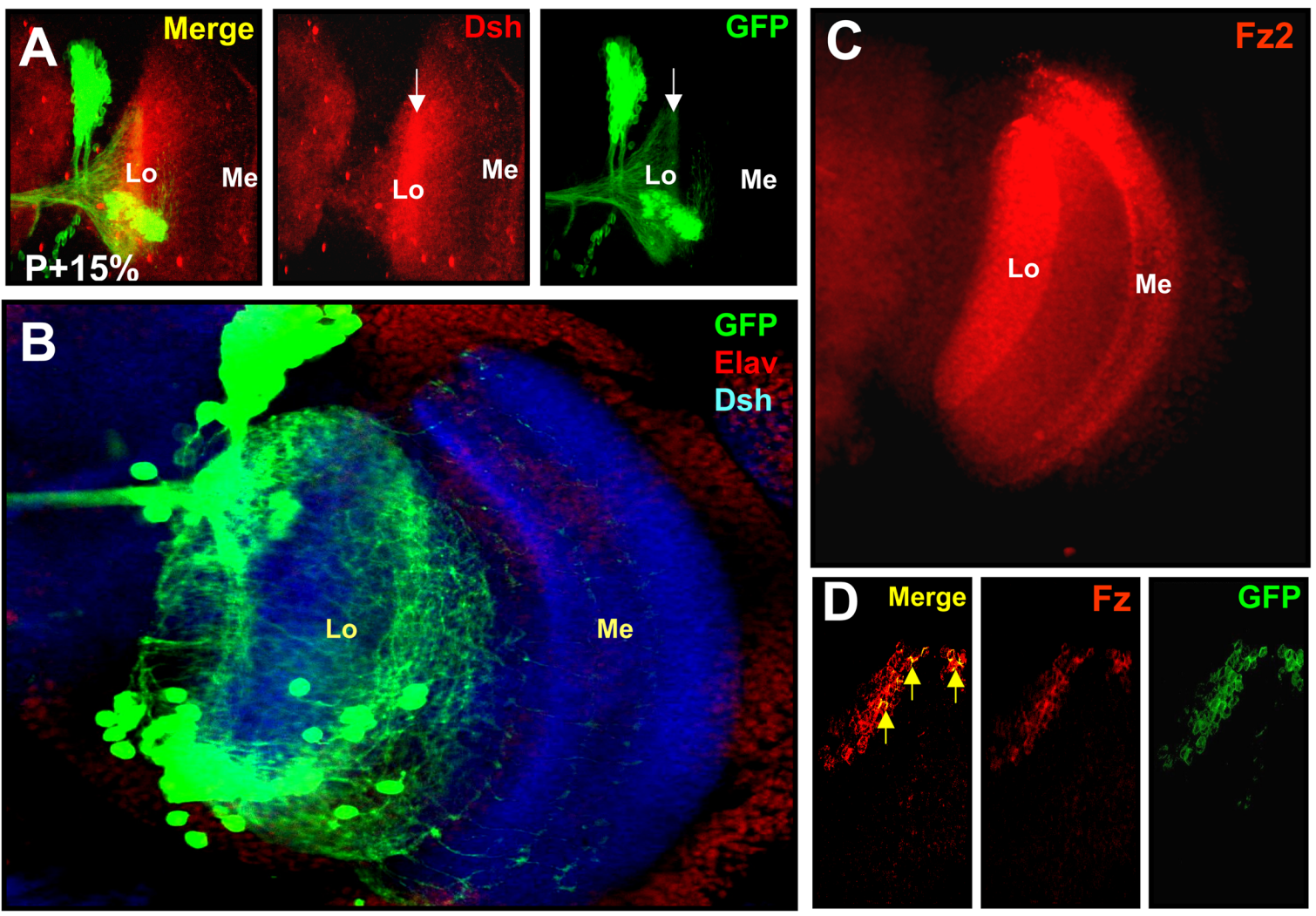

A
Merge
Lo
Me
P+15%
Dsh
Lo
Me
GFP
Lo
Me
B
GFP
Elav
Dsh
Lo
Me
C
Fz2
Lo
Me
D
Merge
Fz
GFP
