# Supplementary material for: A Signaling Network for Patterning of Neuronal Connectivity in the Drosophila Brain
Source: PLoS Biol. 2006 Oct 10;4(11):e348. doi: 10.1371/journal.pbio.0040348 (PMC1592317; doi:10.1371/journal.pbio.0040348)
Supplement: Supplementary file 3 [file pbio.0040348.sg003.pdf]

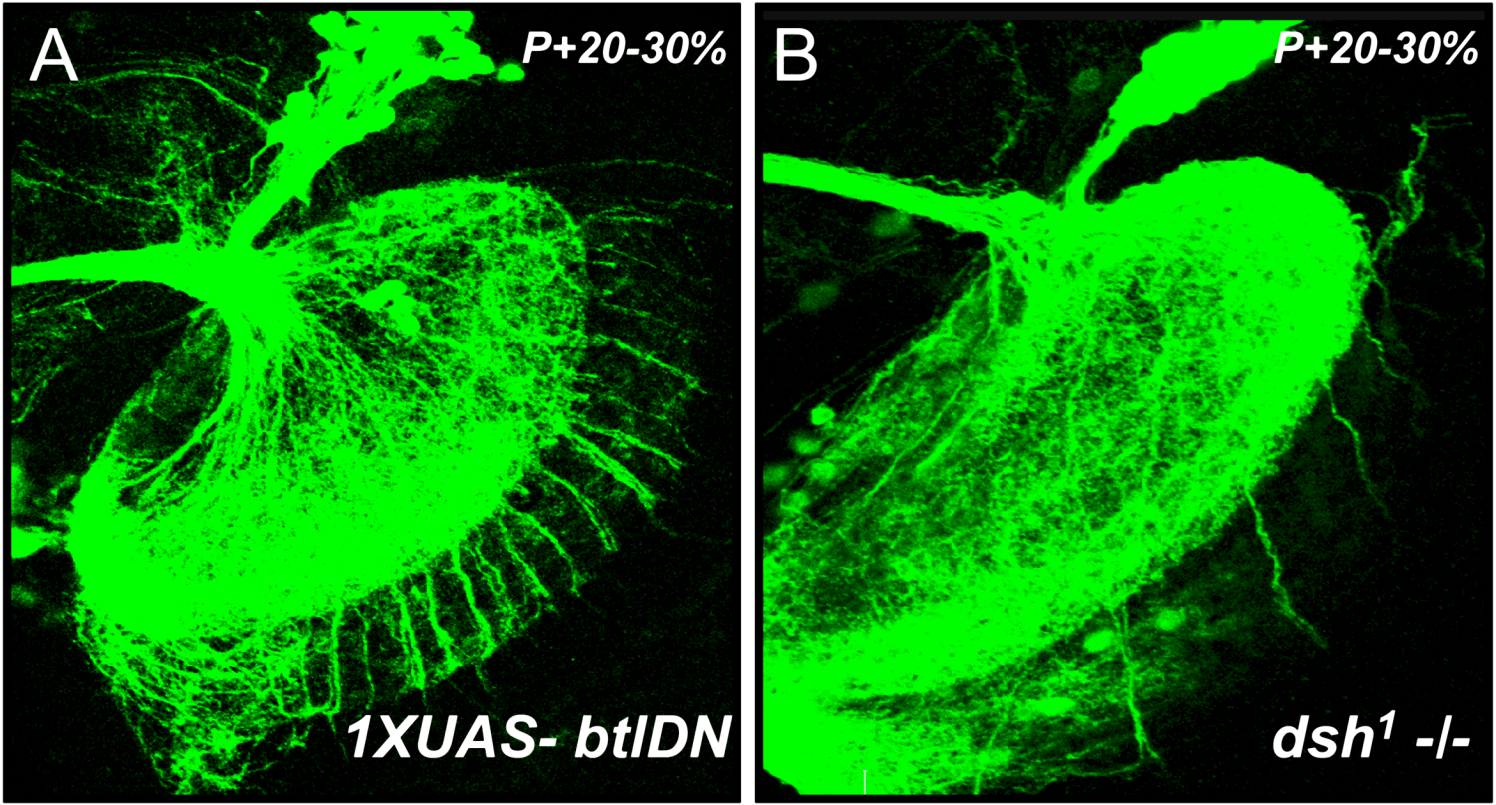
A
P+20-30%
1XUAS- btlDN
B
P+20-30%
dsh1 -/-
